# Connectivity-guided intermittent theta burst versus repetitive transcranial magnetic stimulation for treatment-resistant depression: a randomized controlled trial

**DOI:** 10.1038/s41591-023-02764-z

**Published:** 2024-01-16

**Authors:** Richard Morriss, Paul M. Briley, Lucy Webster, Mohamed Abdelghani, Shaun Barber, Peter Bates, Cassandra Brookes, Beth Hall, Luke Ingram, Micheal Kurkar, Sudheer Lankappa, Peter F. Liddle, R. Hamish McAllister-Williams, Alexander O’Neil-Kerr, Stefan Pszczolkowski, Ana Suazo Di Paola, Yvette Walters, Dorothee P. Auer

**Affiliations:** 1 Mental Health and Clinical Neurosciences, School of Medicine, University of Nottingham, Nottingham, UK; 2 Institute of Mental Health, Nottinghamshire Healthcare NHS Foundation Trust, Nottingham, UK; 3 Clinical Neuromodulation Service, Camden and Islington NHS Foundation Trust, London, UK; 4 Leicester Clinical Trials Unit, University of Leicester, Leicester, UK; 5 Cumbria, Northumberland, Tyne and Wear NHS Foundation Trust, Newcastle upon Tyne, UK; 6 Pennine Care TMS Service, Pennine Care NHS Foundation Trust, Oldham, UK; 7 Northern Centre for Mood Disorders, Translational and Clinical Research Institute, Newcastle University, Newcastle upon Tyne, UK; 8 Centre for Neuromodulation, Northamptonshire Healthcare NHS Foundation Trust, Northampton, UK

**Keywords:** Outcomes research, Magnetic resonance imaging, Depression

## Abstract

Disruption in reciprocal connectivity between the right anterior insula and the left dorsolateral prefrontal cortex is associated with depression and may be a target for neuromodulation. In a five-center, parallel, double-blind, randomized controlled trial we personalized resting-state functional magnetic resonance imaging neuronavigated connectivity-guided intermittent theta burst stimulation (cgiTBS) at a site based on effective connectivity from the right anterior insula to the left dorsolateral prefrontal cortex. We tested its efficacy in reducing the primary outcome depression symptoms measured by the GRID Hamilton Depression Rating Scale 17-item over 8, 16 and 26 weeks, compared with structural magnetic resonance imaging (MRI) neuronavigated repetitive transcranial magnetic stimulation (rTMS) delivered at the standard stimulation site (F3) in patients with ‘treatment-resistant depression’. Participants were randomly assigned to 20 sessions over 4–6 weeks of either cgiTBS (*n* = 128) or rTMS (*n* = 127) with resting-state functional MRI at baseline and 16 weeks. Persistent decreases in depressive symptoms were seen over 26 weeks, with no differences between arms on the primary outcome GRID Hamilton Depression Rating Scale 17-item score (intention-to-treat adjusted mean, −0.31, 95% confidence interval (CI) −1.87, 1.24, *P* = 0.689). Two serious adverse events were possibly related to TMS (mania and psychosis). MRI-neuronavigated cgiTBS and rTMS were equally effective in patients with treatment-resistant depression over 26 weeks (trial registration no. ISRCTN19674644).

## Abstract (web summary)

A randomized controlled trial found that functional connectivity-guided approaches to intermittent theta burst stimulation and repetitive transcranial magnetic stimulation both reduced depressive symptoms in patients with moderate to severe treatment-resistant depression over 26 weeks.

## Main

Antidepressants and psychotherapies are effective for moderate to severe major depressive disorder (MDD)^1^. However, a proportion of individuals with MDD have ‘treatment-resistant depression’ (TRD), with 33% of patients in specialist care^2^ and 22% in primary care failing to respond adequately to two trials of antidepressants^3^.

Repetitive transcranial magnetic stimulation employs strong magnetic pulses to alter activity in neural circuits in the brain implicated in the pathophysiology of depression. High-frequency rTMS to the left dorsolateral prefrontal cortex (lDLPFC) is one of the protocols most commonly used in MDD^4–6^. TBS uses bursts of magnetic pulses mimicking endogenous theta rhythms that may induce plasticity in more distal brain areas^7^. A meta-analysis confirmed the effectiveness and safety of both rTMS and TBS for TRD^4,6,8^. The multicenter THREE-D clinical trial showed that shorter duration of administration iTBS was noninferior to longer-duration rTMS applied to the lDLPFC in the reduction of depression symptoms up to 12 weeks after treatment, but there are no data on longer-term follow-up^9^.

The Federal Drug Administration in the United States approved rTMS for depression in 2008 following confirmation of its effectiveness in a worldwide, 23-site, randomized clinical trial (RCT)^10^. The National Institute for Health and Care Excellence approved TMS for MDD and TRD in the National Health Service in England in 2015 (ref. ^8^). Although more widely used in mental health services across North America, there is patchier implementation in routine mental health practice in other areas of the world. In England, TMS is available in only one in seven mental health services and has not been recommended for use in some countries such as France, albeit based on a questionable review of the evidence^11^. Therefore, the evidence base to date has not been sufficiently convincing to result in widespread implementation or regulatory support in specialist mental health services internationally. One reason for this may be that the effects on TRD are seen as short lived because of the paucity of evidence from large, high-quality RCTs with sufficient duration of follow-up^4,6,8^.

The brain can be subdivided into networks of regions that serve separable functions, and brain connectivity changes as detected by resting-state functional MRI (rsfMRI) can individualize neurostimulation therapy of MDD^12^. TMS stimulation of the lDLPFC (a key node of the central executive network (CEN)) may modulate key nodes within the salience network and default mode network (DMN), leading to rebalancing of abnormal functional connectivity (calculated by correlation of blood oxygenation level-dependent time courses using rsfMRI from different regions of the brain) between and within these networks^13^. However, there is individual variation in the functional connectivity of the lDLPFC to these nodes^14^, suggesting that a personalized approach to targeting the site of delivery of TMS might improve either response rates or the duration of response compared with a single, standardized site of stimulation that is widely used in clinical practice with TMS for depression. Two small RCTs of personalized and accelerated rTMS or iTBS, based on functional connectivity between the subgenual anterior cingulate cortex and lDLPFC, resulted in greater responses in depression over 3–4 weeks versus standardized or sham TBS^15,16^.

A disruption of the reciprocal loop between the DLPFC and insula (a key node of the salience network) has been found in depression^17^, so the insula may represent another target for personalized neuromodulation. A RCT in 27 healthy volunteers found that iTBS delivered to a connectivity-guided target in the lDLPFC with maximum negative influence from the right anterior insula (rAI) improved frontal–insula connectivity^18^. In a small pilot RCT of 18 patients with TRD comparing cgiTBS with connectivity-guided repetitive transcranial stimulation (cgrTMS), the response rate (50% decrease in depression symptoms) showed a statistically insignificant increase from 1 to 3 months in the cgiTBS group but decreased in the cgrTMS group^19^. In both treatment groups, where TMS/TBS stimulation was personalized using effective connectivity (a type of functional connectivity in which directionality is inferred from time-shift analysis of the regional time series^20^), the balance of influence between rAI and lDLPFC was predictive of improvement following a course of TMS^19^. These findings suggested that cgiTBS, personalized based on maximal effective connectivity from rAI to lDLPFC, might lead to longer-lasting efficacy than standard-site rTMS, permitting people with TRD potentially to remain well for longer. However, data are needed with longer follow-up than previously conducted.

The BRIGhTMIND trial was a multicenter, parallel-group, double-blind, randomized, controlled trial. Our primary clinical hypothesis was that rsfMRI-neuronavigated cgiTBS, based on effective connectivity from the rAI to the lDLPFC, would be more efficacious in reducing depression symptoms over 8, 16 and 26 weeks compared with structural MRI-neuronavigated rTMS delivered at the standard stimulation site (F3 of the 10–20-electrode location nomenclature) in patients with TRD. Although a standard site for rTMS was used, the location of that F3 site was personalized using structural MRI. The primary mechanistic hypotheses utilizing fMRI were: (1) baseline effective connectivity from rAI to lDLPFC, or that the balance of influence between these two regions would moderate, or be associated with, improvement in depression symptoms over 26 weeks; and (2) reduction in functional connectivity between the lDLPFC and left dorsomedial prefrontal cortex (lDMPFC) would be associated with improvement in depression symptoms as found in both our pilot work and another study^21^.

## Results

### Patient disposition

**Fig. 1:**
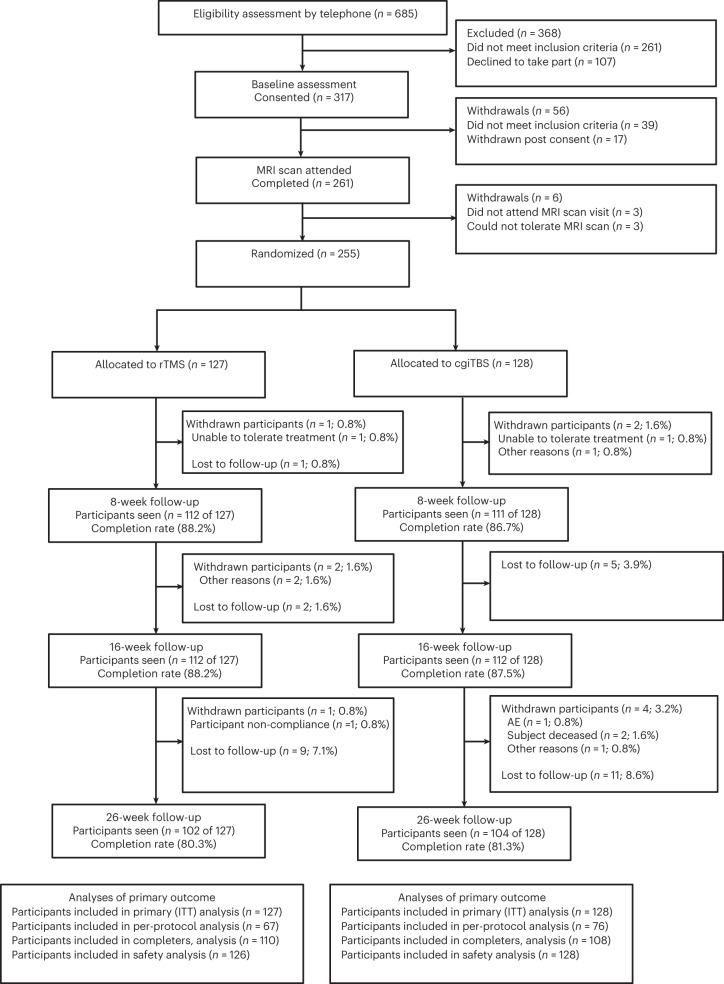
Flowchart of participants through the trial. CONSORT diagram of all participants who were assessed for eligibility for the trial, randomised to repetitive transcranial magnetic stimulation or connectivity guided intermittent theta burst stimulation and followed up to 26 weeks.

**Extended Data Fig. 1:**
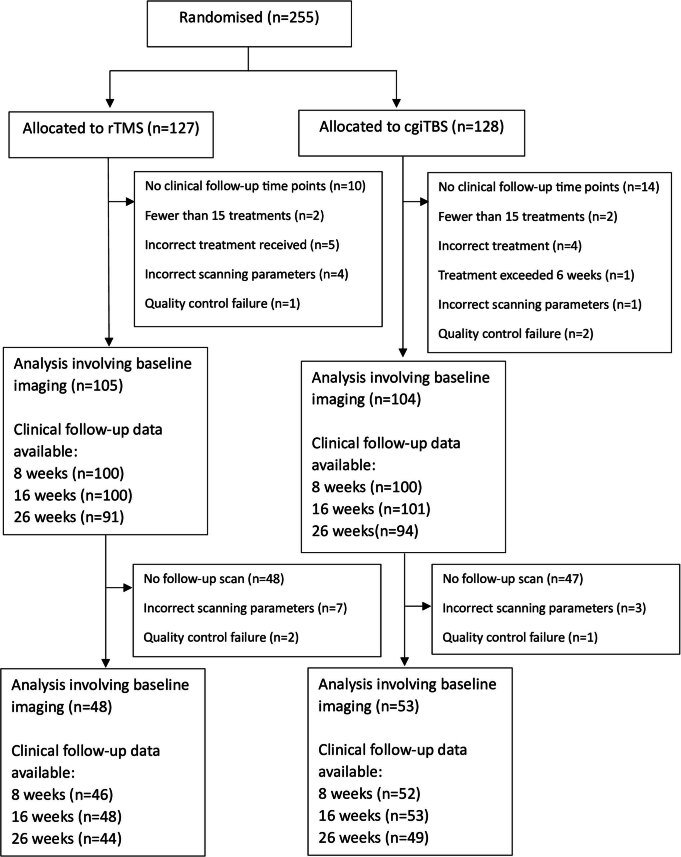
Flow of participants through fMRI protocol. rTMS, repetitive transcranial magnetic stimulation cgiTBS, connectivity guided intermittent theta burst stimulation.

Between 22 January 2019 and 31 January 2022, 685 individuals were identified and completed the initial telephone eligibility screening for the BRIGhTMIND trial (Fig. 1). Recruitment to the study was temporarily suspended between 30 April and 1 August 2020 due to the COVID-19 pandemic. A total of 317 participants consented to the trial, with 39 of these not meeting inclusion criteria and 23 withdrawing between baseline and randomization. A total of 255 participants were randomized, 127 to rTMS and 128 to cgiTBS, with all randomized participants included in the intention-to-treat (ITT) population. In total, 235 participants completed all 20 TMS sessions (92.8%; two participants each in the rTMS and cgiTBS groups discontinued their involvement in the trial altogether during treatment). Comparable completion rates were also found for rTMS versus cgiTBS at 8 weeks (rTMS, 112 out of 127, 88.2% versus cgiTBS, 111 out of 128, 86.7%), 16 weeks (rTMS, 112 out of 127, 88.2% versus cgiTBS, 112 out of 128, 87.5%) and 26 weeks (rTMS, 102 out of 127, 80.3% versus cgiTBS, 104 out of 128, 81.3%). The final follow-up assessment was completed on 3 August 2022. In line with the prepublished analysis plan^22^ for the 255 participants who completed baseline structural and rsfMRI scans and started TMS, 209 (82.0%) were included in the image analysis. Of 114 participants who completed baseline and 16-week follow-up scans, 101 (88.6%) were analyzed (Extended Data Fig. 1).

There were two unintentional unblindings of an outcome assessor and one of a principal investigator to a participant’s treatment. In terms of researchers’ guesses, the majority of treatment allocation predictions were that of ‘don’t know’, with overall rates of 84.8, 79.5 and 74.3% at 8, 16 and 26 weeks, respectively (Extended Data Table 1).

**Extended Data Fig. 2:**
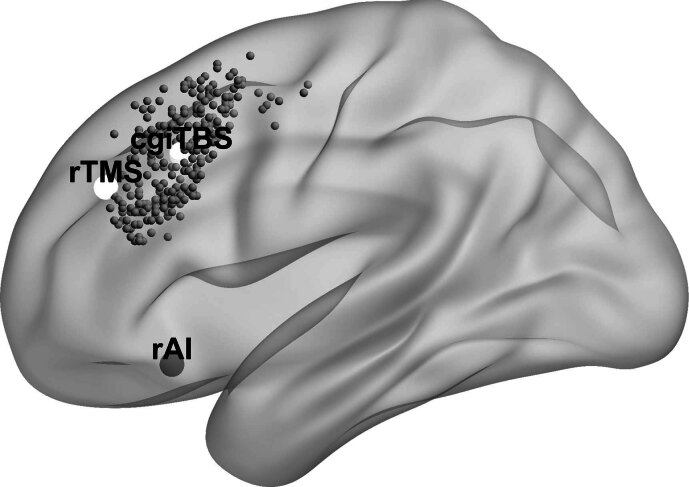
Sites of TMS stimulation. Illustration of transcranial magnetic stimulation (TMS) targets, and regions of interest (ROIs) for the baseline brain connectivity analyses. Small dots indicate the connectivity guided intermittent theta burst stimulation (cgiTBS) left dorsolateral prefrontal cortex (DLPFC) target co-ordinates for individual participants – these are the locations with maximal effective connectivity from the right anterior insula (rAI) as a large grey dot. The mean cgiTBS target across participants is shown as a large white dot. The mean repetitive transcranial magnetic stimulation (rTMS) target is shown as a large white dot; this is the F3 target for the group that received rTMS. Plotted with BrainNet Viewer (Xia et al.^71^) on the smoothed ICBM152 atlas.

At baseline the mean age of participants was 43.7 years (s.d. 14.0), with 132 (51.8%) women and 232 (91%) of white ethnicity (Table 1). The median duration of current depression episode was 6.1 years (interquartile range (IQR) 2.1, 12.9) and the median number of depressive episodes was two (IQR 1, 4). Ninety-five participants (37.3%) were categorized as high treatment resistance (nonresponse to more than approximately six treatments) on the modified Massachusetts General Hospital Treatment Resistant Depression staging score (MGH^23^), 73 (28.6%) as medium treatment resistance (nonresponse to around four or five treatments) and 87 (34.1%) as low treatment resistance (nonresponse to two or three treatments), with 198 participants (77.6%) currently taking antidepressants. The mean baseline scores on the primary outcome variable—the GRID version of the 17-item Hamilton Depression Rating Scale (GRID-HDRS-17; ref. ^24^)—were 23.9 (s.d. 4.7) for the rTMS group and 22.9 (s.d. 4.7) for the cgiTBS group (Tables 2 and 3). An inter-rater reliability assessment of outcome assessors, completed across treatment centers, showed an intraclass coefficient of 0.94 between GRID-HDRS-17 scores, with a 95% reference interval for the difference (between any pair of raters) of 0.66–0.99. Across both treatment groups the median distance between the intended stimulation point on the scalp and the actual stimulation point, or between the actual stimulation point on the first, and subsequent, sessions, was about 0.5 cm, and median angle difference was about 7° (Extended Data Table 2; sites of stimulation are shown in Extended Data Fig. 2).

**Table 1:** Baseline characteristics of participants

Characteristics	rTMS (*n* = 127)	cgiTBS (*n* = 128)
Age (years) Mean (s.d.)	43.8 (13.1)	43.7 (15.0)
Gender (*n*(%))		
Men	65 (51.2%)	58 (45.3%)
Women	62 (48.8%)	70 (54.7%)
Ethnicity (*n*(%))		
White British	106 (83.5%)	108 (84.4%)
White Irish	4 (3.1%)	1 (0.8%)
Other White	6 (4.7%)	7 (5.5%)
White and black African	1 (0.8%)	2 (1.6%)
White and Asian	1 (0.8%)	0 (0%)
Other mixed	1 (0.8%)	2 (1.6%)
Indian	4 (3.1%)	2 (1.6%)
Pakistani	2 (1.6%)	2 (1.6%)
Bangladeshi	0 (0%)	1 (0.8%)
Other Asian	0 (0%)	1 (0.8%)
Black Caribbean	1 (0.8%)	0 (0%)
Chinese	0 (0%)	1 (0.8%)
Other ethnic group	1 (0.8%)	1 (0.8%)
Marital status: married/cohabiting (Yes, *n*(%))	76 (59.8%)	55 (43.0%)
Dependants (children/other) (Yes, *n*(%))	42 (33.1%)	36 (28.1%)
Employment/education (*n*(%))		
Full-time	39 (30.7%)	37 (28.9%)
Other employment	36 (28.3%)	26 (20.3%)
Retired	13 (10.2%)	17 (13.3%)
Unemployed	39 (30.7%)	48 (37.5%)
Receipt of benefits (Yes, *n*(%))	52 (40.9%)	45 (35.2%)
Duration of current major depressive episode (months)	117	122
Median (IQR)	69.7 (27.9, 129.0)	79.3 (24.9, 163.3)
Number of depressive episodes	84	91
Median (IQR)	2.0 (1.0, 4.0)	1.0 (1.0, 4.0)
Baseline CTQ (respondents)	120	120
Mean (s.d.)	47.1 (17.4)	45.1 (16.2)
Number of participants with treatment history	127	128
Category of baseline MGH treatment-resistant depression score (*n*(%))		
Low, 2.0–3.5	42 (33.1%)	45 (35.2%)
Medium, 4–6	36 (28.3%)	37 (28.9%)
High, ≥6.5	49 (38.6%)	46 (35.9%)
Baseline medication use		
Antidepressants	94 (74.0%)	104 (81.3%)
Tricyclic antidepressants	10 (7.9%)	11 (8.6%)
MAOIs	1 (0.8%)	0 (0%)
SSRIs	41 (32.3%)	46 (35.9%)
SNRI	31 (24.4%)	39 (30.5%)
Other^a^	34 (26.8%)	36 (28.1%)
Antidepressant combination	22 (17.3%)	19 (14.8%)
Antipsychotic augmentation	19 (15.0%)	23 (18.0%)
Lithium augmentation	3 (2.4%)	11 (8.6%)
Methylphenidate augmentation	0 (0%)	1 (0.8%)
Modafinil augmentation	0 (0%)	1 (0.8%)
Triiodothyronine augmentation	0 (0%)	3 (2.3%)
Hypnotics/sleeping tablets	7 (5.5%)	9 (7.0%)
Anxiolytics	7 (5.5%)	7 (5.5%)
Electroconvulsive therapy during current episode of depression (*n*(%))	6 (4.7%)	4 (3.1%)

MAOIs, monoamine oxidase inhibitors; SSRIs, selective serotonin reuptake inhibitors; SNRIs, serotonin and norepinephrine reuptake inhibitors.

^a^Other baseline medication refers to the following antidepressants: trazadone, bupropion, mirtazapine, reboxetine, agomelatine or vortioxetine). The number of current major depressive episodes has been set to missing for participants whose number was entered as 99. The duration of current major depressive episodes was calculated using the date of randomization and start date of the episode.

**Table 2:** Primary and secondary outcome scores and response to treatment

Measure	Baseline		8 weeks		16 weeks		26 weeks		cgiTBS versus rTMS over 26 weeks	
	rTMS *n* mean (s.d.)	cgiTBS *n* mean (s.d.)	rTMS *n* mean (s.d.)	cgiTBS *n* mean (s.d.)	rTMS *n* mean (s.d.)	cgiTBS *n* mean (s.d.)	rTMS *n* mean (s.d.)	cgiTBS *n* mean (s.d.)	Adjusted mean difference (95% CI)^a^	*P* value
GRID-HDRS-17 primary analysis^b^	127	128	127	128	127	128	127	128		
	23.9 (4.7)	22.9 (4.7)	15.6 (s.e. 0.7; 95% CI 14.2, 17.0)	14.5 (s.e. 0.6; 95% CI 13.2, 15.7)	15.9 (s.e. 0.8; 95% CI 14.5, 17.5)	15.3 (s.e 0.7; 95% CI 13.9, 16.7)	16.1 (s.e. 0.8; 95% CI 14.5, 17.8)	14.9 (s.e 0.7; 95% CI 13.4, 16.3)	−0.31 (−1.87, 1.24)	0.689
BDI-II	127	128	111	109	109	110	99	102		
	34.4 (8.9)	32.3 (8.8)	23.5 (12.6)	21.3 (10.7)	24.7 (12.2)	22.3 (12.4)	23.6 (12.6)	21.6 (12.0)	−0.54 (−2.90, 1.82)	0.653
PHQ-9	127	128	111	109	109	109	99	102		
	20.2 (4.6)	19.4 (4.4)	13.4 (7.5)	12.3 (6.3)	13.8 (7.2)	13.5 (7.3)	13.7 (7.6)	13.1 (7.5)	−0.12 (−1.54, 1.30)	0.871
GAD-7	127	128	111	109	109	108	99	102		
	13.3 (4.7)	13.1 (4.6)	9.3 (6.3)	8.9 (4.9)	9.3 (5.5)	9.1 (5.3)	9.9 (6.1)	8.9 (5.6)	−0.19 (−1.24, 0.86)	0.726
WSAS	127	128	111	109	109	109	99	102		
	29.0 (6.8)	27.6 (7.8)	22.1 (10.9)	21.2 (9.5)	22.2 (10.7)	22.4 (10.2)	22.2 (10.7)	21.5 (10.8)	0.60 (−1.39, 2.59)	0.554
EQ-5D-5L VAS	127	128	111	109	109	109	98	102		
	43.0 (19.3)	43.4 (17.1)	52.8 (21.0)	54.7 (18.8)	53.2 (20.2)	56.7 (19.4)	53.8 (21.2)	55.8 (20.5)	1.98 (−1.96, 5.91)	0.325
THINC-it									cgiTBS versus rTMS baseline and 16-week adjusted mean difference (95% CI)^c^	*P* value
CRT response time (ms)	123	127	NA	NA	76	72	NA	NA		
Missing	4	1								
	717.67 (238.55)	708.05 (249.25)			606.28 (183.02)	614.12 (210.71)			−1.48 (−55.05, 52.09)	0.957
DSST total correct	122	127	NA	NA	76	72	NA	NA		
Missing	5	1								
	51.17 (18.26)	49.15 (21.18)			55.76 (16.93)	52.36 (19.59)			−4.25 (−8.56, .062)	0.053
N-back total correct	122	126	NA	NA	76	72	NA	NA		
Missing	5	2								
	22.82 (10.81)	21.56 (9.66)			25.28 (9.25)	24.56 (9.63)			−1.27 (−3.51,0.97)	0.264
TMT response time (s)	123	126	NA	NA	76	72	NA	NA		
Missing	4	2								
	30.08 (15.99)	34.70 (25.26)			27.11 (16.34)	31.07 (25.10)			21.03 (−3.28, 45.33)	0.090
PDQ-5-D score	123	127	NA	NA	75	71	NA	NA		
Missing	4	1								
	12.89 (4.47)	13.29 (4.46)			10.55 (5.07)	10.83 (94.79)			0.43 (−0.57, 1.43)	0.394

CRT, based on a choice reaction task; DSST, based on the digit symbol substitution test; N-back, based on the one-back paradigm TMT, which is based on part B of the trails-making task; PDQ-5, subjective perceived deficits questionnaire, five domains. NA, not available.

^a^Adjusted for: treatment center (stratification variable), baseline HDRS-17 score and degree of treatment-resistant depression (minimization variables) and treatment arm with participant ID as the random effect. Secondary continuous clinical outcomes models were also adjusted for their respective baseline measure.

^b^Standard errors along with 95% CIs are reported for the GRID-HDRS-17-estimated means at follow-up time points, because multiple imputation was used to perform the primary analysis.

^c^Cognition outcomes were the dependent variables, with the independent variables of interest being the THINC-it time point (baseline and 16 weeks), treatment group (rTMS and cgiTBS), baseline GAD-7, baseline HDRS-17 and change in HDRS-17 score between baseline and week 16. Models also included three interaction terms: treatment group × time point, time point × change in HDRS-17 and change in HDRS-17 × treatment group × time point. Confounder variables included age, gender, site and MGH group. Any confounder variable not found to be significant on initial testing was removed from the models and the analysis rerun.

**Table 3:** Proportions of responders, sustained responders and remitters in rTMS and cgiTBS groups

Measure	Rate in each treatment arm (*n*/total (%))		cgiTBS versus rTMS (adjusted odds ratio (95% CI))^a^	*P* value
	rTMS	cgiTBS		
Responders				
8-week follow-up	35/112 (31.3)	39/111 (35.1)	1.13 (0.63, 2.03)	0.682
16-week follow-up	38/112 (33.9)	39/112 (34.8)	1.03 (0.57, 1.87)	0.916
26-week follow-up	31/102 (30.4)	36/104 (34.6)	1.18 (0.63, 2.20)	0.615
Sustained responders				
16-week follow-up	23/127 (18.1)	28/128 (21.9)	1.21 (0.64, 2.29)	0.557
26-week follow-up	22/127 (17.3)	29/128 (22.7)	1.40 (0.74, 2.66)	0.307
Remitters				
8-week follow-up	19/112 (16.9)	23/111 (20.7)	1.09 (0.53, 2.26)	0.818
16-week follow-up	23/112 (20.5)	23/112 (20.5)	0.84 (0.42, 1.69)	0.631
26-week follow-up	21/102 (20.6)	26/104 (25.0)	1.21 (0.61, 2,41)	0.590

^a^Binary logistic models were used for analysis of responders, remitters and sustained responders, with treatment comparison estimates presented similarly to those reported for primary outcome analysis, with the exception of reporting adjusted odd ratios. Responder (≥50% reduction on GRID-HDRS-17 from baseline), remitter (score of ≤8 on GRID-HDRS-17) and sustained responder (continuing-response ≥50% reduction on GRID-HDRS-17 following response at the previous timepoint).

### Primary outcome

**Fig. 2:**
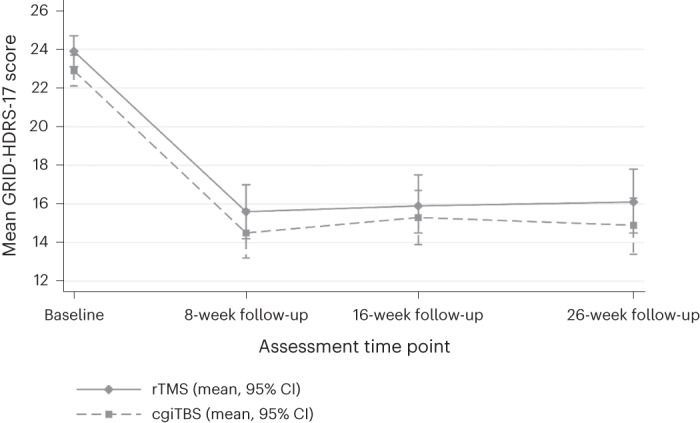
Mean (s.e.) GRID-HDRS-17 scores over time for analysis of primary ITT (multiple imputation).

As shown in Tables 2 and 3, the adjusted mean difference for GRID-HDRS-17 over 26 weeks was not significant and not clinically important (<3-point difference^25^) between rTMS and cgiTBS treatment groups for the primary analysis (−0.31 (95% CI −1.87 to 1.24), *P* = 0.689). At 8 weeks following randomization, both treatment groups showed a clinically substantial decrease (≥7 (ref. ^26^), rTMS 8.3, cgiTBS 8.4) in mean GRID-HDRS-17 scores that were maintained at both 16 weeks (rTMS 8.0, cgiTBS 7.6) and 26 weeks (rTMS 7.8, cgiTBS 8.0; Tables 2 and 3 and Fig. 2).

### Secondary outcomes

There were no significant differences between rTMS and cgiTBS on any of the secondary clinical outcome measures (Tables 2 and 3). At the 26-week follow-up in both groups, 67 (32.5%) of 206 participants were responders (≥50% drop in baseline GRID-HDRS-17 score), 47 (22.8%) of 206 participants were remitters (≥7 on GRID-HDRS-17 score) and 51 (20.0%) of 255 participants were sustained responders (>50% drop in baseline at both 16 and 26 weeks). At 8, 16 and 26 weeks for both treatment groups there were, on average, clinically substantial important improvements in self-rated depression as measured by the Patient Health Questionnaire (PHQ-9 (ref. ^27^), ≥6.0 points^28^) and the Beck Depression Inventory-II (BDI-II^29^, ≥10.0 points^30^), with greater than minimum clinically important improvements in Generalized Anxiety Disorder Assessment (GAD-7)^31^ (≥3.3 points^32^), Work and Social Adjustment Scale (WSAS^33^, >3.7 points^34^) and the Euroqol-5D-5L visual analog scale of overall perceived health (EQ-5D-5L VAS^35^, ≥8.0 points^36^). The cognition analysis showed improvements over time on the Transforming Health with Integrated Care–integrated tool (THINC-it) cognitive battery^37^ for sustained attention (Choice Response Task) (*F*(1, 155.49) = 11.28, *P* = 0.001), executive functioning (Trail-making Task) (*F*(1, 152.45) = 5.50, *P* = 0.020) and working memory (N-back task) (*F*(1, 151.09) = 7.75, *P* = 0.006).

**Extended Data Fig. 3:**
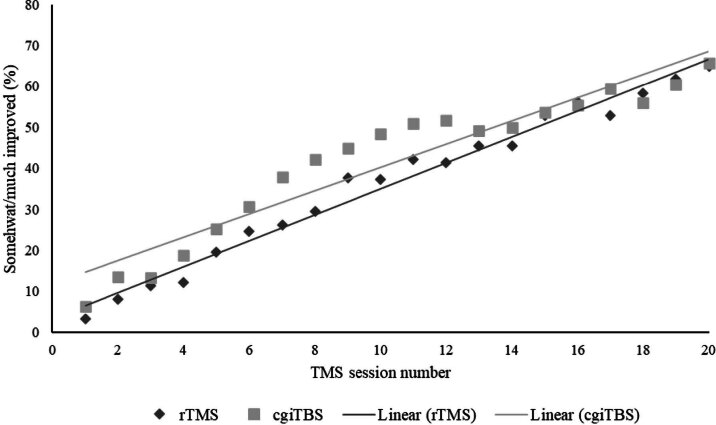
Percentage of participants who felt somewhat or much better over the 20 TMS sessions. TMS, transcranial magnetic stimulation. rTMS, repetitive transcranial magnetic stimulation. cgiTBS, connectivity guided intermittent theta burst stimulation. Participant self-rated as somewhat or much improved on modified 5-point clinical global impression scale.

The participants’ impression of change analysis demonstrated that, at the tenth session, 105 (42.9%) of 245 participants reported feeling somewhat, or much, better. By session 20 this was reported for 155 (65.4%) of 237 participants. The relationship between treatment session number and perceived improvement generally followed a linear trend for both groups, with the proportion experiencing a benefit continuing to increase even at the 19th and 20th sessions (Extended Data Fig. 3).

### Safety

One out of 255 randomized participants was excluded from the safety population because they had experienced a suspected 2-s seizure during the first motor threshold testing and before any treatment had been provided (Table 4). Seventeen further serious adverse events (SAEs) were reported for 12 participants. There were two deaths: one participant had an underlying cardiovascular health condition and died following a myocardial infarction and another died from opiate poisoning, with the coroner’s inquest concluding accidental death. Both participants had completed their course of TMS treatments and died close to the 26-week assessment, with both deaths reported as unlikely to be related to TMS treatments. All further SAEs required hospital admission. Two SAEs were reported as possibly related to TMS treatment (one in each treatment arm): a psychotic episode with severe anxiety and depression 1 month following TMS completion and a manic episode following the 14th treatment session. One participant was admitted to hospital for nausea and vomiting following their baseline MRI scan, with this event reported as probably related to the scan due to the position of the neck while in the MRI scanner. All further SAEs were reported as unrelated to the study. There were a further 17 adverse events (AEs) of self-harm for 11 participants, and two AEs regarding an episode of syncope during treatments. Both participants suspended TMS on that day but completed the remainder of their TMS course without further incident.

**Table 4:** Frequency of SAEs and AEs of self-harm and syncope

Event type	Safety population	
	rTMS, *n* = 126	cgiTBS, *n* = 128
SAE		
Hospitalization for nausea and vomiting	1 (1%)	0
Hospitalization for pulmonary embolus	1 (1%)	0
Hospitalization for COVID-19	0	1 (1%)
Death from accidental opiate poisoning	0	1 (1%)
Hospitalization for investigation of fatigue	0	1 (1%)
Hospitalization for head injury	1 (1%)	0
Hospitalization for headache	1 (1%)	0
Death from myocardial infarction	0	1 (1%)
Hospitalization for high temperature	0	1 (1%)
Hospital admission for anaphylaxis due to insect bites	1 (1%)	0
Hospital admission for chest pains and breathlessness	1 (1%)	0
Hospital admission for low blood pressure	1 (1%)	0
Hospital admission related to pre-existing hidradenitis suppurativa	1 (1%)	0
Hospital admission: psychotic episode with severe anxiety/depression	1 (1%)	0
Hospitalization due to mania episode	0	1 (1%)
Hospitalization for shortness of breath	0	1 (1%)
Voluntary hospital admission for ECT	1 (1%)	0
Suspected seizure before first TMS session^a^	1	0
AE		
Self-harm	5; *n* = 4 (3%)	12; *n* = 7 (6%)
Syncope	2; *n* = 2 (2%)	0

Data are presented as *n*, number of participants affected, and the percentage of participants affected per SAE or AE (for each treatment arm) from the total number of participants randomized. ECT, electroconvulsive therapy.

^a^Not included in the Safety population because the participant did not have any TMS treatment delivered due to experiencing a seizure.

### Exploratory clinical outcomes

Moderator analyses demonstrated that higher baseline GRID-HDRS-17, higher baseline generalized anxiety (GAD-7) and completion of <20 stimulation sessions predicted lesser improvement in depression symptoms over 26-week follow-up (Supplementary Table 1). However, interactions between treatment arm and these moderator variables (as well as gender) were not statistically significant.

### Neuroimaging outcomes

**Extended Data Fig. 4:**
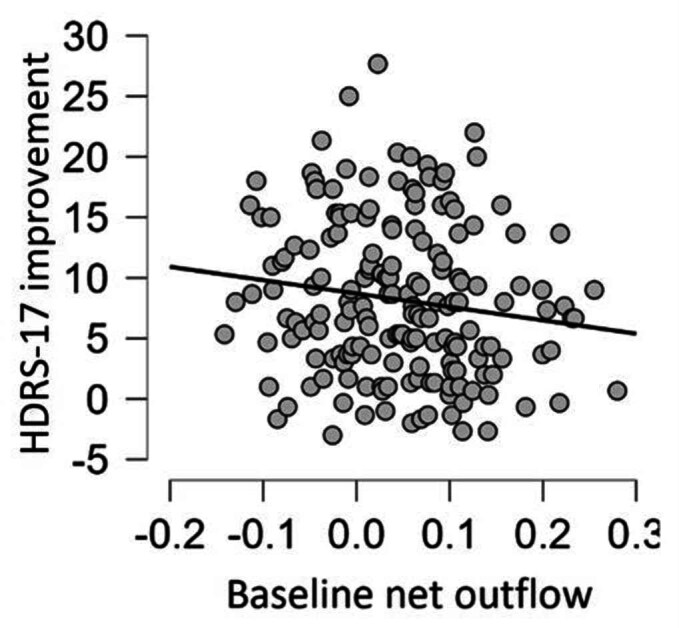
Reduction in baseline net outflow from right anterior insula to left dorsolateral prefrontal cortex in relation to mean improvement in GRID-HDRS-17 score over 26 weeks. Reduction in GRID version of the 17=item Hamilton Depression Rating Scale (GRID-HDRS-17) total score (improvement) from baseline to follow-up, collapsed across follow-up time points, as a function of baseline net outflow from the right anterior insula (RAI) seed region to the connectivity guided intermittent theta burst stimulation (cgiTBS) target region within the left dorsolateral prefrontal cortex (DLPFC). Each data point is a participant. Lower net outflow (that is, lesser influence of the RAI on the left DLPFC and greater influence of the left DLPFC on the RAI) was associated with greater improvement.

The primary neuroimaging hypothesis—that baseline effective connectivity from rAI to lDLPFC would predict clinical improvement—was not supported for GRID-HDRS-17, BDI-II or PHQ-9 scores (*P* > 0.1, 185–201 participants included across time points). However, baseline rAI net outflow (effective connectivity from rAI to lDLPFC minus that from lDLPFC to rAI) was supported for GRID-HDRS-17 (main effect of net outflow: *F*(1,196) = 4.04, *P* = 0.046). Enhanced improvement was associated with greater positive influence from lDLPFC to rAI and lesser positive influence from rAI to lDLPFC (Extended Data Fig. 4). This relationship did not differ between treatment groups or across post-treatment time points. Baseline net outflow was less positive in 16-week HDRS-17 responders than in nonresponders across both treatment groups (*t*(199) = 2.022, *P* = 0.044).

**Extended Data Fig. 5:**
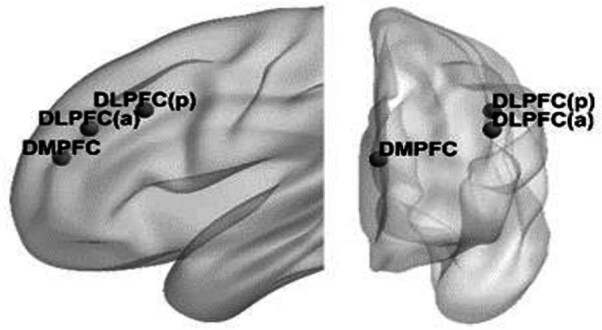
Regions of interest for change in brain connectivity analysis for dorsolateral and dorsomedial prefrontal cortex. Regions of interest (ROIs) for the change in brain connectivity analyses, plotted on a standard brain image (smoothed ICBM152 atlas), in the sagittal (left) and coronal (right) planes. DMPFC: dorsomedial prefrontal cortex; DLPFC(a): anterior DLPFC site; DLPFC(p): posterior DLPFC site.

**Extended Data Fig. 6:**
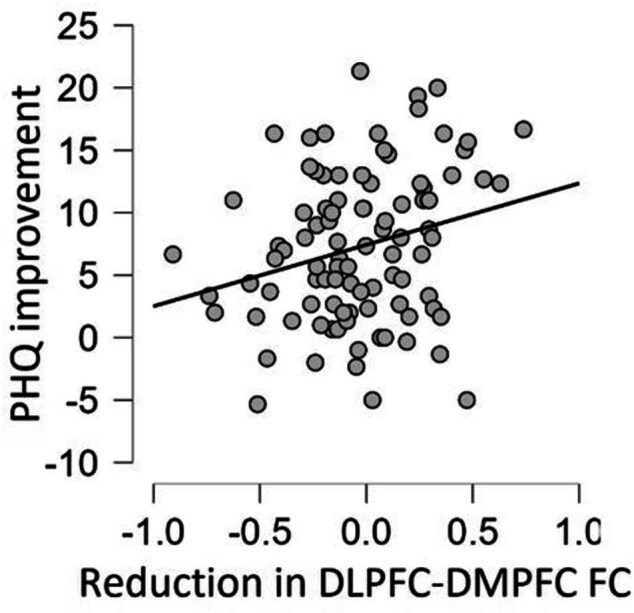
Reduction in left dorsolateral prefrontal cortex to left dosrsomedial prefrontal cortex functional connectivity with improvement in PHQ-9 score over 26 weeks. Reduction in 9-item Personal Health Questionnaire (PHQ-9) total score (improvement) from baseline to follow-up, collapsed across post-treatment time point, as a function of reduction in functional connectivity between the left dorsomedial prefrontal cortex (DMPFC) region and the posterior left dorsolateral prefrontal cortex (DLPFC) region. Each data point is a participant. Greater clinical improvement was associated with greater reduction in functional connectivity between these regions.

Reduction in functional connectivity between lDLPFC and lDMPFC from baseline to 16 weeks was not supported for change in GRID-HDRS-17 for either the anterior or posterior DLPFC site specified in the protocol (*P* > 0.1, 93–101 participants included across time points) but was significant for improvements in PHQ-9 (main effect of change in functional connectivity; PHQ-9: *F*(1,105.6) = 6.89, *P* = 0.010; Extended Data Figs. 5 and 6) and approached significance for improvements in BDI-II (*F*(1,104.4) = 4.81, *P* = 0.031) for the posterior lDLPFC site. These relationships did not differ across groups, suggesting a direct link between network change and antidepressant effect.

### Sensitivity analyses

**Fig. 3:**
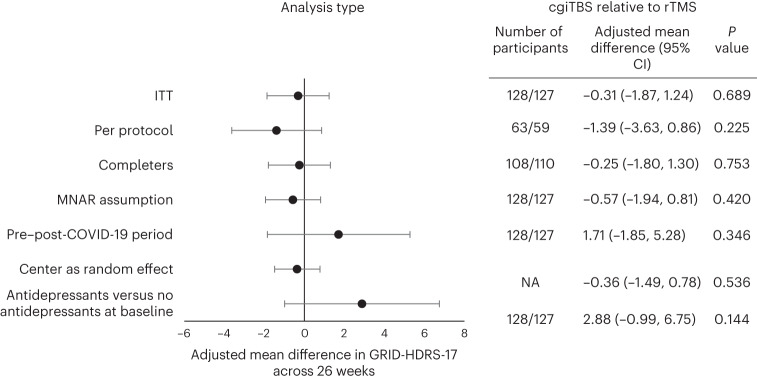
ITT and sensitivity analyses of primary outcome measure following cgiTBS relative to rTMS.

Differences between treatment arms were not significant for any of the sensitivity analyses conducted; no center effects and no effects of being on no antidepressants at baseline were observed (Fig. 3 and Extended Data Table 3). Deviations leading to participant exclusion in the per-protocol analysis are given in Extended Data Table 4.

### Post hoc analyses

To further understand the net outflow results, we examined whether baseline effective connectivity from lDLPFC to rAI alone predicted improvement on either GRID-HDRS-17 or HDRS-6 (6-item Hamilton Depression Rating Scale) score^38,39^. The relationship was nonsignificant for GRID-HDRS-17 (*P* = 0.280) but significant for HDRS-6 (greater positive influence predicted greater improvement; *F*(1,197) = 4.21, *P* = 0.042].

## Discussion

The BRIGhTMIND study is a large, adequately powered trial in the United Kingdom using TMS and iTBS for TRD with outcomes at 26 weeks. There were no statistically significant or clinically important differences observed between cgiTBS and rTMS on primary and secondary clinical outcomes across 26 weeks, demonstrating that cgiTBS did not show superior clinical efficacy compared with structural MRI-neuronavigated rTMS. Both treatment arms demonstrated clinically substantial improvements in the primary outcome of observed depression and self-rated measures of depression, with above-minimum clinical important changes in self-rated anxiety, functioning and quality of life. For both treatment arms around one-third of participants showed a response, one-fifth achieved remission and one-fifth demonstrated a sustained response for 6 months. The results are encouraging given that two-thirds of participants were classed as medium to high treatment-resistant depression (approximately equivalent to failure to respond to four or more antidepressants), with a long duration of current depressive episode (median 6 years).

The two RCTs closest in design to ours are the THREE-D^9^ and THETA-DEP trials^40^, both of which compared iTBS versus rTMS using structural MRI neuronavigation, the former with follow-up over 12 weeks and the latter for 26 weeks. Response and remission rates for depression, and improvements in anxiety and quality of life up to 26 weeks in the present trial, are consistent with THETA-DEP, a single-site study of only 60 participants with a shorter duration of current depression episode (mean 20 months) and less treatment resistance^40^. Thus, while previous evidence suggests that the beneficial effects of rTMS on mood in TRD may be relatively short lived, lasting only 1–3 months^5,9^, both MRI-neuronavigated TMS protocols in our study and THETA-DEP led to sustained responses maintained for >6 months post treatment in one in five participants. The current results demonstrate this finding in an adequately powered sample, and with more persistent and difficult-to-treat depression than previously described. We do not know whether such sustained responses would occur with non-MRI-neuronavigated TMS.

Magnetic resonance imaging-guided neuronavigation may be advantageous in terms of reduced coil drift and off-target placement compared with traditional elastic cap scalp targeting^41^, with our study demonstrating that, in the vast majority of cases, the site of stimulation of TMS varied by <1 cm and the angle of stimulation by <10° from the target site over the course of 20 sessions. Although one previous MDD study reported greater clinical efficacy for MRI-guided neuronavigated TMS versus scalp-based targeting methods^42^, others found no difference in clinical efficacy^43,44^. These studies focused on immediate rather than longer-term efficacy. Because previous RCTs of iTBS^5^ measured response and remission only immediately following treatment, the benefits of MRI-guided neuronavigated versus non-navigated iTBS following initial treatment are unknown. Therefore, future research might compare the clinical efficacy and cost effectiveness for MRI-neuronavigated TMS versus non-neuronavigated TMS over longer-term follow-up given the additional cost of MRI scans.

The THREE-D study offered up to 30 TMS sessions for a number of participants and demonstrated slightly higher rates of response (40–50%) and remission (20–30%) than our study^9^. Taking this into consideration, with the proportion of participants in our trial feeling somewhat (or, much) better and still increasing at the 19th and 20th treatment sessions, outcomes might be further enhanced in those participants who are still improving with up to 30 TMS treatments. Both treatments were associated with improvements over time for sustained attention, executive functioning and working memory, consistent with the conclusion of a recent meta-analysis that rTMS has modest cognitive-enhancing effects in MDD^45^.

Our fMRI findings are supportive of the longer-term benefits of both cgiTBS and rTMS, with some putative evidence of a normalizing effect of brain dysconnectivity. People with MDD show increased positive connectivity between the CEN and DMN on rsfMRI while in healthy controls these networks are anticorrelated or uncorrelated^46^. Our resting fMRI analysis suggests reduction in functional connectivity between baseline and 16 weeks between the posterior lDLPFC (part of CEN) and lDMPFC (part of DMN), consistent with the hypothesis that restoration of normal anticorrelation may be associated with improvements in depression. Despite the different proximities of the targets to the posterior lDLPFC (close for cgiTBS, distant for rTMS), the findings were similar between the two treatment arms, replicating our unpublished pilot work and a previous study^21^—although only in self-rated measures of depression. If independently confirmed, TMS-induced restoration of the normal CEN–DMN anticorrelation pattern may be a putative (direct or indirect) mechanism of its antidepressant efficacy—at least for some response domains. Improvement with TMS might indicate a reduction in intrusion of DMN-related, internal-world processing and rumination on CEN-related external-world processing and task performance, and might be consistent with the finding of attentional lapses in people with MDD^47^. Such changes may be better captured by the self-report BDI-II and PHQ-9 measures, which measure poor concentration and subjective processing, rather than the GRID-HDRS-17 measure, which does not directly measure these processes^38,39^.

We found that the imbalance of influence between rAI and lDLPFC (‘net outflow’) predicted improvement in depression symptoms over 26 weeks across both treatment groups. Reduced baseline net outflow from rAI to lDLPFC was associated with response on GRID-HDRS-17 at 16 weeks in both treatment groups. Post hoc analysis suggested that improvement in core symptoms of depression was associated with dominant baseline effective connectivity from the lDLPFC on the rAI. A putative mechanistic explanation requiring further research is that greater influence of lDLPFC on rAI might enable the effects of TMS to spread more effectively from the lDLPFC to rAI, thereby enhancing its neuromodulatory effect on the insula.

One strength of the RCT is the multicenter design. The sample was large, with diversity in age, ethnicity and other demographic features. In comparison with the clinical population of TRD where there is a greater proportion of females, there was equal representation of men and women. Otherwise, the sample is generalizable to clinical populations in the United Kingdom with TRD. Treatment resistance was verified from both patient accounts and clinical records, although this might have been underestimated if patient recall and records were incomplete. The measurement of treatment resistance also did not include psychological treatments, which are often accessible in England. There was a high rate (93%) of treatment completion of all 20 TMS sessions and follow-up (average 85%). From the neuronavigation data, TMS treatment was delivered with a high level of precision in relation to the MRI-derived coordinates and varied little in either site or other TMS parameters across 20 sessions, except for slight adjustments in positioning or motor threshold according to predetermined criteria. Inter-rater reliability checks suggested that measurement of the primary outcome was comparable between centers. Blinding of the intervention was successful for observers of outcome. A key aspect of the trial was the active input of people with lived experience of depression and transcranial magnetic stimulation (the BRIGhTMIND Lived Experience Advisory Group), who informed all aspects of the design, conduct and interpretation of the trial.

The study was, however, highly disrupted and suspended for 6 months by public health measures put in place to control the COVID-19 pandemic. With the input of our Lived Experience Advisory Group and external review by the Independent Trial Steering and Data Monitoring and Ethics Committee, we made a number of substantial changes to the protocol including (1) a change in primary outcome from response at 16 weeks to average change over 8, 16 and 26 weeks, (2) a revised power calculation and (3) a move from face-to-face to remote assessment of outcome where possible^48,49^. In light of the public health emergency, the study would not have been completed without these changes given the resources available for it. Pre–post COVID analysis of outcomes did not show any clinically important or statistically significant effect of the pandemic. Response rates at 16 weeks, the original primary outcome, show very little difference between treatment groups. It is very unlikely that the changes made to the trial through necessity on account of the COVID-19 pandemic made any material difference to any outcome or conclusion from the trial.

Limitations included that, although TMS treatments were well matched for number of pulses per treatment session, session duration and number of sessions, they differed in stimulation frequency (iTBS or rTMS), intensity of stimulation (80% resting motor threshold (RMT) or 120% RMT) and approach to selection of treatment location (resting state effective connectivity versus structural MRI). Previous RCTs suggest that iTBS and rTMS may be equivalent in efficacy in TRD^9,40^. There is some uncertainty about the importance of the intensity of stimulation but, in the current trial, 120% RMT was not tolerated well by a minority of trial participants, with a reduction in intensity required in such participants in the rTMS arm to limit dropout from treatment. The reduction in intensity of stimulation was important because the number of treatment sessions moderated the mean reduction in depression symptoms over 8, 16 and 26 weeks in both the current RCT and THREE-D RCT^50^. The importance of the approach to selection of treatment location is unknown. To match the treatment arms for the number of pulses per treatment session, we introduced an accelerated form of iTBS with five runs of 600 pulses over 37 min in each treatment session with approximately 5-min intervals of nonstimulation between runs. Dosage and time intervals between TBS protocols could affect meta-plasticity, either by reducing or reversing the effect of synaptic plasticity or by increasing the effect of synaptic plasticity^51–53^. In some individuals, 5-min intervals between iTBS runs might increase short-term cortical inhibition, reducing the effectiveness of the whole iTBS session^52^ and thereby making a group difference between cgiTBS and rTMS more difficult to demonstrate. It is worth noting that the current results are comparable in terms of response to the THETA-DEP RCT^40^ over 26 weeks for both the iTBS and rTMS groups, so the additional runs after the first run of 600 pulses may have had little additional efficacy in the cgiTBS treatment group.

Given these limitations, there are several ways of interpreting the results of the current study—that cgiTBS is not superior to rTMS. The most probable explanation is that precise targeting of the lDLPFC–rAI loop is unimportant in terms of the clinical or mechanistic efficacy of TMS. This would be consistent with the notion that spatially distinct targets may modulate the same or overlapping brain circuits. Alternatively, one could posit that the frontoinsular loop and its interaction with the DMN are irrelevant for the treatment effect. We consider this less likely for two reasons: (1) the net outflow from lDLPFC–rAI moderated primary outcome in both treatment groups, suggesting that lDLPFC–rAI functional connectivity might play some role in the TMS response; and (2) connectivity between the posterior lDLPFC, closely matching the average cgiTBS target, and dmPFC was associated with subjective improvement ratings. We cannot rule out that the 80 versus 120% RMT strength biased efficacy somewhat towards rTMS, but we did not see delayed subject response trajectories in cgiTBS. Taken together, the lack of difference on any clinical or fMRI measure, with similar effects on functional connectivity, supports the interpretation that precision targeting of TMS treatments may not be advantageous at the group level using nonaccelerated rTMS or the current accelerated iTBS protocol. Future tertiary analyses of the rich multimodal data will explore possible subgroup-specific clinical and neuroplasticity effects.

A key clinical finding was the duration of TMS effects up to 26 weeks with both treatment groups. However, the interpretation of these results is hampered by the lack of a sham treatment group and the lack of an end-of-treatment measure of primary outcome at 6 weeks. In relation to the latter, there was only a 2-week gap to the first follow-up assessment, with negligible changes to other treatment in that period. Our Lived Experience Advisory Panel (LEAP) group advised that measurement of outcome at 6 and 8 weeks would be burdensome, so we chose the 8-week outcome measurement rather than 6-week to measure effects seen at 1 month following TMS in our pilot study. There are three possible explanations for the long duration of TMS in this RCT: (1) a lasting effect of TMS; (2) a nonspecific treatment effect due to regression to the mean, expectancy, hope or structure to the day; or (3) the effects of additional drug treatment for depression and anxiety, particularly at 16 and 26 weeks. A meta-analysis of placebo responses in RCTs of rTMS in TRD reported response and remission rates of 20 and 11% at the end of treatment^54^ versus 33 and 19%, respectively, in BRIGhTMIND. A high degree of treatment resistance (all failed two treatments, the majority four) and long duration of current illness (median 6 years) are associated with lower placebo responses with treatments for depression, including TMS^55–58^. In a RCT of a similar sample of participants with a comparable duration of current depression, largely recruited from the highest-recruiting site in BRIGhTMIND, the remission rate at 26 weeks was only 12% (ref. ^59^).

The occurrence of fMRI changes associated with treatment in the study does not exclude a placebo response, especially given the finding of an overlap between brain regional activity modified by placebo and TMS for targets including lDLPFC^60^. Nevertheless, one study showed that the placebo response did not impact on connectivity with the rAI^61^, as seen with rTMS/cgi TBS in the present study. Some of the changes in outcome in both groups, especially at 16 and 26 weeks, may have been due to alterations in medication. For clinical and ethical reasons in this severe TRD group, changes in antidepressant or other medication were allowed and were made in 19% of the sample by 16 weeks. Exclusion of such participants in the per-protocol analysis, or being on an antidepressant or not at baseline, did not result in statistically significant or clinically important differences in primary outcome between treatment groups. Taken together, TMS is likely to have had a substantial impact on the duration of response but some of that change is due to nonspecific effects and clinically indicated medication changes, as would be the case in regular clinical care. How much of the change was due to TMS could be established only by an adequately powered RCT comparing iTBS or rTMS versus sham control on depression symptoms over 26 weeks. We proposed such a RCT to our funders when we first sought funding for the BRIGhTMIND study, but such a design was rejected for clinical and ethical reasons in such a severe, vulnerable group of patients. Therefore, it may not be possible to carry out such a trial.

In conclusion, this study found that cgiTBS and MRI-neuronavigated rTMS are equally effective and safe. Patients showed clinically substantial improvements in depression that were sustained up to 26 weeks. These findings raise the possibility that some TRD patients unresponsive to other treatments could be kept well, while many others would derive clinically significant benefits, from one or two MRI-navigated courses of 20 (or possibly more) iTBS or rTMS sessions over a year.

## Methods

### Study design and participants

Participants were recruited from primary and secondary care settings at five treatment centers across UK National Health Services (NHS): Nottinghamshire Healthcare NHS Foundation Trust; Northamptonshire Healthcare NHS Foundation Trust; Cumbria, Northumberland, Tyne and Wear NHS Foundation Trust; Camden and Islington NHS Foundation Trust; and Pennine Care NHS Foundation Trust. The treatment centers were chosen to reflect geographical diversity and the fact that some had previous experience of TMS. The trial design and methods are outlined in two published trial protocols^48,49^.

A participant met inclusion criteria if they were: aged ≥18 years; met criteria for DSM-V major depressive disorder using a structured clinical interview^4,62,63^; had moderate to severe depression defined as a score of 16 or more on the GRID version of GRID-HDRS-17 (ref. ^24^)); had TRD defined as scoring 2 or more on MGH^23^, which was adapted for new treatment options (Supplementary Information^48^); and had the capacity to provide informed consent before any trial-related activities.

Participants were excluded if they had: a history of bipolar disorder or depression secondary to other mental disorder; neurological conditions—for example, brain neoplasm, cerebrovascular events, epilepsy, neurodegenerative disorders or previous brain surgery; standard contraindications to MRI (for example, irremovable metal objects in and around body, pregnancy, red tattoos on the head, neck and back or claustrophobia); major unstable medical illness requiring further investigation or treatment; in 2 weeks before baseline assessment any change in prescribed medication, treatment with lamotrigine, gabapentin or pregabalin, or intermittent benzodiazepines (or daily prescription >5-mg diazepam equivalents) or hypnotics >7.5-mg zopiclone equivalent; current substance abuse or dependence (DSM-5 criteria^62^); previous TMS treatment; high risk of suicidality; potential complicating factors for TMS treatment (for example, hairstyles impeding close coil placement, piercings); involved with any other clinical trial at the time of consent or 6 months previously; or unable to read or understand English.

Participants were recruited through specialist mental health services across the five treatment centers and neighboring NHS trusts near the treatment centers, self-referrals and through patient identification centers recruiting through primary care services.

A questionnaire was used to telephone prescreen interested participants, with potentially eligible participants invited to attend a baseline assessment with an outcome assessor. At the baseline assessment all participants gave written informed consent and study eligibility was determined by the outcome assessors using SCID-5-RV, GRID-HDRS-17 and MGH. Furthermore, to assist with determination of study eligibility, medical and psychiatric history—including a detailed assessment of treatment resistance—was obtained from primary care notes and secondary care mental health service case files where available. Participants also completed the childhood trauma questionnaire (CTQ^64^) and self-report sociodemographic information was collected. All assessments were completed face to face at the hospital sites before the COVID-19 pandemic, which were then changed to video conferencing or telephone methods. Participants also completed a baseline MRI assessment with scans used to derive personalized treatment targets, and for a mechanism-of-action analysis with MRI at baseline and 16 weeks.

### Ethics approval

The clinical trial received research ethics committee approval and health research authority approval from the East Midlands Leicester Central Research Ethics Committee (no. 18/EM/0232). Research design and execution included local scientists at each site and was shared with all local sites.

### Randomization and masking

Participants were randomly assigned in a 1:1 ratio to rTMS or cgiTBS. The TMS staff delivering treatment conducted the randomization process via a web-based randomization system (Sealed Envelope, www.sealedenvelope.com) immediately before the start of the participant’s first treatment session. Randomization was stratified by study site and minimized on severity of depression (GRID-HDRS-17: score 16–23, moderate or ≥24, severe) and degree of treatment resistance (low 2–3.5, medium 4–6, high ≥6.5), as assessed at the baseline assessment. Treatment allocation was conveyed only to TMS administration staff at each site via email.

Participants, referring clinical teams and outcome assessors were kept blinded with respect to treatment allocation until after the participant’s final follow-up assessment. Any unintended unblinding of outcome assessors was recorded, with other assessors completing all further assessments for that participant. At each follow-up assessment the outcomes assessor was asked to guess the participant’s treatment allocation.

### Procedures

A total of 3,000 pulses were delivered in each rTMS or cgiTBS session, which was around 38 min in duration for the purposes of blinding participants and assessors of outcome.

A 70-mm, figure-of-eight coil (E-z Cool coil) and a Magstim Horizon Performance Stimulator with StimGuide Navigated TMS Package (Magstim Co.) was used for all rTMS and cgiTBS treatments. Twenty once-daily sessions were delivered per participant over a 4–6-week period for both treatment arms.

Participants assigned to cgiTBS received 50-Hz bursts of three pulses (80% resting motor threshold), with bursts repeated every 200 ms (5 Hz). Bursts were presented in 10-s cycles consisting of 2 s of stimulation and 8 s of rest; there were 20 such cycles per run (600 pulses per run). Five runs were presented per session, with 5-min inter-run intervals (3,000 pulses per session). The cgiTBS brain target was defined based on Granger Causality Analysis as the location within the lDLPFC receiving maximal effective connectivity from the rAI (Montreal Neurological Institute coordinates: *x* = 30 mm, *y* = 24 mm, *z* = −14 mm, determined using the participant’s rsfMRI and T1-weighted structural MRI scans)^48,49^. The StimGuide Navigated TMS Package computed the nearest location for stimulation on the scalp from an individualized head model based on structural MRI and three fiducial points: the nasion and left preauricular and right preauricular sites.

Participants assigned to rTMS followed the standard US Food and Drug Administration-approved protocol^10^. Stimulation was at 120% resting motor threshold with 75 × 4-s trains of 10 Hz interspersed by 26-s intertrain intervals, with a total of 3,000 pulses per session. The rTMS brain target was determined using the participants’ structural MRI to target a standard Montreal Neurological Institute coordinate *x* = −41 mm, *y* = 43 mm, *z* = 32 mm (selected a priori as the parenchymal voxel closest to the F3 site in a standard brain). As with the cgiTBS treatment arm, the StimGuide Navigated TMS Package was used to compute the stimulation site from the same individualized head model and the three fiducial points mentioned above.

Motor threshold (percentage) was determined at the first treatment session and determined again on the sixth treatment session for both treatment arms. Standardized steps were developed for participants who were unable to tolerate the cgiTBS or rTMS protocols, which involved either movement of the site of stimulation by 1 cm from the MRI-derived coordinates or a reduction in motor threshold.

Outcome data from assessment scales (GRID-HDRS-17, BDI-II^29^), PHQ-9 (ref. ^27^)), GAD-7 (ref.^31^)), WSAS^33^), EQ-5D-5L^35^) and EQ-5D-5L VAS^35^ were collected at baseline and at 8, 16 and 26 weeks following randomization. An adapted version of the client service receipt inventory^65^ was collected at baseline and at 16- and 26-week follow-ups. Participants completed MRI scans at baseline and within 2 weeks of the 16-week follow-up assessment. The THINC-It tool^37^ for cognition was originally collected at baseline assessment and at all three follow-up time points. However, following the COVID-19 pandemic the THINC-It tool was collected at the baseline and 16-week MRI scans only. To assess participants’ beliefs about the efficacy of treatment, and based on the advice of our patient and public involvement (PPI) representatives, we adapted the seven-point patient global impression of change measure^66^ to a shortened, five-point Likert scale (1–5, much worse to much better, with as many rating points for worse and improved mental state). Patient acceptability was also assessed with a purposively designed five-point Likert measure rated from 1 to 5: unacceptable (negative effects outweigh benefits) to acceptable (beneficial effects outweigh negative effects). These two measures were assessed at every TMS session and at each follow-up time point. Before the COVID-19 pandemic, participants were given the option to complete follow-up assessments face to face or remotely, with all subsequently being completed by telephone or video conferencing during and after the COVID-19 pandemic. Travel expenses were covered for participation in the study, along with a £10.00 shopping voucher at 16- and 26-week follow-up assessments, as a mark of respect and gratitude for the time and input of the participants to the follow-up aspects of the trial. Participants recruited later in the study completed the Quick Inventory of Depressive Symptomatology Self-Rated version^67^ at baseline and 8, 16 and 26 weeks for the purposes of a substudy on cognition and fMRI (Supplementary Information^48^). Therefore, this measure should not be regarded as a secondary outcome of the trial and is not reported here.

### Outcomes

The primary clinical outcome measure was mean change across 8, 16 and 26 weeks in depression symptoms from baseline using GRID-HDRS-17. HDRS-17 is the most frequently used observer-rated measure of depression for RCTs of treatments for depression^68^, and the GRID form was utilized given evidence of improved inter-rater reliability^24^.

Secondary clinical outcomes were mean changes from baseline over 26 weeks on BDI-II, PHQ-9, GAD-7, WSAS and EQ-5D-5L VAS; mean changes from baseline to 16 weeks for the five cognitive tasks in the THINC-It tool; mean changes from baseline to 8, 16 and 26 weeks separately on GRID-HDRS-17; proportion of responders at 8, 16 and 26 weeks (defined as a reduction of ≥50% on GRID-HDRS-17 from baseline); proportion of remitters at 8, 16 and 26 weeks (defined as a score of ≤8 on GRID-HDRS-17); proportion of sustained responders at 16 and 26 weeks (defined as a continuing-response ≥50% reduction on GRID-HDRS-17 following response at the previous timepoint); and patient global impression of change at each TMS session and each follow-up time point and adverse events (side effects) checklist after each TMS session.

Magnetic spectroscopy, cost effectiveness outcomes (EQ-5D-5L and adapted client service receipt inventory), acceptability outcomes (five-point, purposively designed Likert scale and qualitative interviews) and further safety outcomes (common and uncommon side effects) will be reported separately.

### Monitoring of adverse events

Internationally agreed definitions for AEs and SAEs were adopted and applied^69^. Seizures were reported as SAEs. Syncope was recorded as an AE unless the participant was admitted to hospital, in which case it was defined as a SAE. Any participant found to be at risk to themselves (suicide, neglect) or others, or developing a SAE, was referred to the relevant clinical services. A review by a clinical expert in TRD was offered to any participant whose depression had become more severe at 16- and 26-week follow-ups, for safety reasons.

### Role of the LEAP

The LEAP was a panel of PPI representatives with lived experience of depression, some of whom had additional personal experience of TMS, that informed all aspects of the design, development and running of the BRIGhTMIND trial. The LEAP was chaired by an experienced PPI organizer (P.B.) and included representatives from all centers. Efforts were made to ensure inclusivity by gender, ethnic background and personal experience. LEAP members were paid for their time. Specific recommendations from the LEAP were: the completion of 20 TMS sessions over 6 weeks from 4 weeks; outcomes measured only at 8 weeks rather than at 6 and 8 weeks because of the burden on participants; travel buddies came to MRI and TMS appointments; all research materials were rewritten with lay and inclusive language, leading to a doubling of study website hits; advertising at specific sites to promote inclusivity—for example, places of worship to recruit people of South Asian origin; and adaptation of the patient global impression of change. Further changes were suggested by the LEAP to ensure continuation of the trial during the COVID-19 pandemic, such as the use of staff photographs and profiles while wearing masks during face-to-face and remote appointments.

### Changes due to the COVID-19 pandemic

Substantial amendments to the protocol made in light of the COVID-19 pandemic have been reported in a trial protocol publication^2^. These changes were made in response to national and local public health measures in respect to the COVID-19 pandemic, with the approval of each site’s clinical research governance organizations, the sponsor (Nottinghamshire Healthcare NHS Foundation Trust), the LEAP, the Trial Management Group, the Independent Trial Steering Committee and Data Management and Ethics Committee and the funders. As one of the public health measures in the COVID-19 pandemic, these changes did not require NHS Ethics and Health Research Authority approval. The study was suspended, except for remote follow-up assessment, from 19 March to 1 August 2020. The following key changes were made from 1 August 2020 to the end of the study: (1) all baseline clinical assessments, obtaining written and informed consent and all follow-up clinical assessments, were made remotely by video conferencing supported by telephone and email. (2) All MRI, TMS treatment and THINC-it assessments were conducted face to face, with COVID-19 pandemic precautions reducing the maximum throughput of participants in the trial. (3) THINC-it assessments were conducted only at baseline and 16 weeks, alongside MRI scans, and were not conducted at 8 and 26 weeks—no other changes were made to assessments although there was loss of follow-up MRI scans at 16 weeks from 19 March to 1 August 2020. (4) The primary outcome was changed from response at 16 weeks to average change in total HDRS-17 score at 8, 16 and 26 weeks. (5) Sample size was reduced from 368 to 266 participants given the slower recruitment rate, because of the pandemic precautions. (6) One site did not reopen once the study reopened because of the loss of staff required to conduct the TMS and research assessment, and was replaced by another site. (7) The analysis plan was changed to reflect the change of the primary outcome variable and to add a pre–post-COVID sensitivity analysis. (8) Further funding was obtained to address the period of suspension of the study and slower recruitment rate.

### Sample size calculation

The National Institute for Health and Care Excellence defined three points as a clinically important difference in outcome on HDRS-17 for depression disorders^25^. We compared the mean change in depression symptoms from baseline over 26 weeks in the cgiTBS group with that in the rTMS group. Assuming a standard deviation of 8 in the mean difference between groups, as informed by our pilot work^19^ and a previous randomized controlled trial in chronic persistent depressive disorder^59^, a sample size of 266 participants would provide 89.3% power to detect a mean difference of three points in GRID-HDRS-17 over 26 weeks between the groups at the 5% two-sided significance level, assuming a correlation between follow-up measures of 0.7 and 20% data loss/dropout.

### Statistical analysis

A statistical analysis plan was published before the primary analysis was undertaken and provides further detail on the analysis^70^. The primary analysis of primary outcome was conducted on the ITT population (all participants randomized to treatments), with the multiple-imputation technique being implemented to deal with missing data in instances where participants were missing GRID-HDRS-17 scores. Gender, ethnicity, age, center, baseline GRID-HDRS-17 score and degree of TRD were used as predictors of primary outcome to substitute the missing data with the predicted values from a multivariate normal regression equation. A total of 20 imputations were estimated. A mixed linear regression model was utilized, which adjusted for center (stratification variable), baseline GRID-HDRS-17 score and baseline MGH score (minimization variables), visit number and a categorical variable for treatment arm (rTMS arm as reference). Participant ID was included as random effect. The treatment comparison estimate is presented as adjusted mean difference between the treatment arms, with two-sided 95% CIs and *P* values and statistical significance considered at ≤5%. Analysis of secondary clinical outcomes was performed in a similar way, conducted on the ITT population using an available-data approach. Binary logistic models were used for analyses of responders, remitters and sustained responders, with treatment comparison estimates presented similarly to those reported for primary outcome analysis except for reporting of adjusted odds ratios. Participants randomly assigned to treatment and who completed at least one TMS session were included in the safety population.

Secondary analyses of primary outcome included a per-protocol analysis (excluding participants with major protocol violations: if original treatment protocols were not administered; 20 treatment sessions were completed after 6 weeks; more than 4 days had elapsed between treatments; MDD pharmacotherapy or psychotherapy changed before 16-week follow-up) and a completersʼ analysis (that is, participants completed ten or more sessions of rTMS or cgiTBS delivered to the correct MRI coordinates and assessed at baseline and 16 weeks). Sensitivity analyses of primary outcome included a missing-not-at-random (MNAR) assumption analysis using a control-based imputation approach and a pre–post-COVID-19 period analysis.

Stata (v.16) was used for all data analyses except for cognition outcomes, which were analyzed in IBM SPSS statistics (v.25). With regard to neuroimaging, a protocol was published before receipt of any clinical data or statistical analysis^22^. Preprocessing steps were as detailed in that protocol. Mixed-effects models were implemented in SPSS (v.18) and JASP (0.18) software and estimated with restricted maximum likelihood. Participant served as the random effect with a scaled identity variance–covariance matrix, with the dependent variable being clinical improvement from baseline in GRID-HDRS-17 (primary outcome measure), PHQ-9 or BDI-II (planned exploratory outcome measures). In addition to baseline connectivity, or change in connectivity, relevant to a given hypothesis we included as independent variables the post-treatment time point (8, 16 or 26 weeks) and treatment group (rTMS or cgiTBS), and the interaction of connectivity with either or both variables. Age, gender, MGH treatment-resistance group, GAD-7, CTQ and study group site were explored as potential confounding variables; where these were nonsignificant they were removed from the model. MGH group and site were significant for baseline connectivity analyses; no confounders were significant for analyses examining change in connectivity. Reduction in GAD-7 was significantly associated with reduction in measures of depression from baseline to follow-up, but this did not change the significance of reported findings. The threshold of significance was set at the 5% level for each of our prespecified analyses. Due to the use of two prespecified regions of interest for lDLPFC in analyses examining change in functional connectivity between DLPFC and DMPFC, Holm–Bonferroni correction was applied for two tests across *P* values for each term (apart from the confounder variables) of that mixed model. In preplanned sensitivity analyses excluding patients for whom the cgiTBS target lay outside the left middle frontal gyrus according to the Harvard–Oxford cortical atlas at 10% threshold, only two such cases were identified and there was minimal change in statistical parameters. A nonpreplanned exploratory analysis was performed with HDRS-6 (ref. ^38^) which, unlike GRID-HDRS-17, is a unidimensional measure of depression over time^39^, to further understand the effects of baseline net rAI to lDLPFC outflow results in relation to depression symptoms over 26 weeks.

The BRIGhTMIND trial was registered with the ISRCTN registry (no. ISRCTN19674644) on 2 October 2018, amended on 18 September 2020 to account for COVID-19, and is now registered as complete.

### Reporting summary

Further information on research design is available in the Nature Portfolio Reporting Summary linked to this article.

## Online content

Any methods, additional references, Nature Portfolio reporting summaries, source data, extended data, supplementary information, acknowledgements, peer review information; details of author contributions and competing interests; and statements of data and code availability are available at 10.1038/s41591-023-02764-z.

### Supplementary information

Supplementary Information Supplementary Table 1.

Reporting Summary

## Figures and Tables

**Extended Data Table 1:**
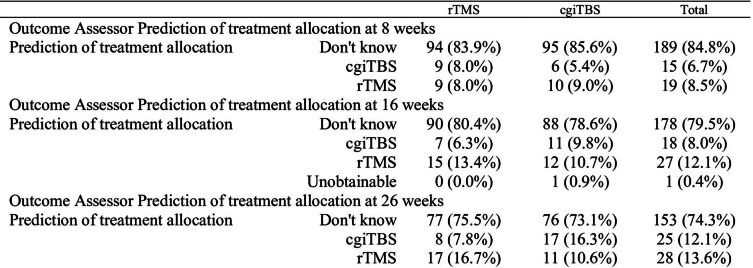
Outcome assessor predictions of treatment allocation

rTMS, repetitive transcranial magnetic stimulation cgiTBS, connectivity guided intermittent theta burst stimulation.

**Extended Data Table 2:**
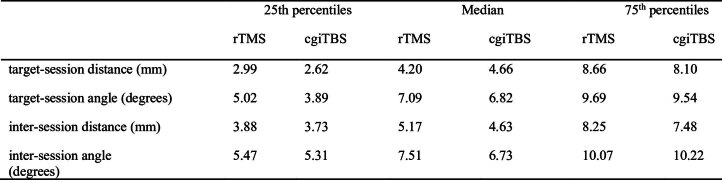
Target-session and inter-session distance and angle of TMS

TMS, transcranial magnetic stimulation. rTMS, repetitive transcranial magnetic stimulation cgiTBS, connectivity guided intermittent theta burst stimulation.

**Extended Data Table 3:**
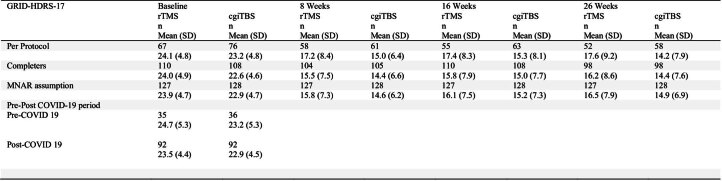
GRID-HDRS-17 descriptive statistics for the secondary and sensitivity analyses of the primary outcome

GRID-HDRS-17, GRID version of 17-item Hamilton Depression Rating Scale. rTMS, repetitive transcranial magnetic stimulation. cgiTBS, connectivity guided intermittent theta burst stimulation. MNAR, missing not at random.

**Extended Data Table 4:**
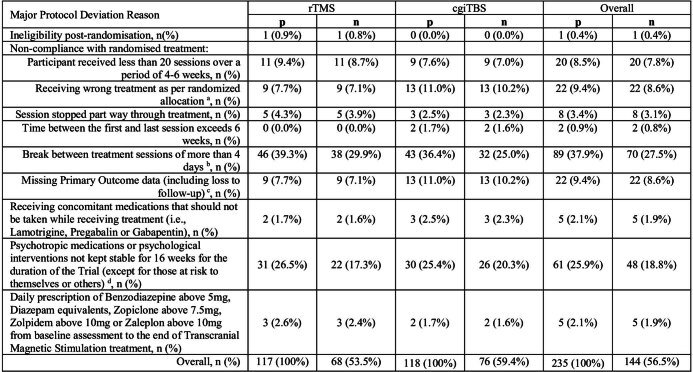
Breakdown of major protocol deviation reasons by deviation type and number of participants affected by deviation type

p: number of protocol deviations by deviation type, n: number participants affected per deviation type Please note the percentage corresponding to the proportion of participants affected per major deviation type (including the total for each treatment arm and overall) was calculated out of the total number of participants randomised. rTMS, repetitive transcranial magnetic stimulation. cgiTBS, connectivity guided intermittent theta burst stimulation.

^a^TMS treatment was delivered for part of a treatment session or course at the incorrect coordinates in relation to the magnetic resonance imaging (MRI) data or at the incorrect motor threshold in the transcranial magnetic stimulation standardized operating procedure. There were no instances of the wrong treatment allocation being delivered.

^b^A deviation of this type was added to the summary table above for participants that had a break of more than 4 days between TMS sessions and did not have a protocol deviation recorded for this on the Protocol Deviations Case Report Form of the study.

^c^Defined as the failure to provide the GRID version of the 17-item Hamilton Depression Rating Scale (GRID-HDRS-17) score at all the follow-up assessment time points: 8, 16 and 26 weeks.

^d^Changes in psychotropic medications were captured under the category of ‘Other Deviation’ and they were adjudicated by the Chief Investigator as to determine whether or not they were classed as this type of medication.

## Data Availability

We shall make data available to the scientific community with as few restrictions as feasible while retaining exclusive use until the publication of major outputs. Anonymized data, including all the trial data published in this manuscript, will be deposited at the University of Nottingham data repository (https://rdmc.nottingham.ac.uk) to encourage wider use.

## Code availability

The computer code used to calculate the coordinates for cgiTBS or rTMS stimulation from fMRI and structural MRI scans in the BRIGhTMIND study can be found at https://github.com/SPMIC-UoN/brightmind_pipeline.
