# Supplementary material for: Connectivity-guided intermittent theta burst versus repetitive transcranial magnetic stimulation for treatment-resistant depression: a randomized controlled trial
Source: Nat Med. 2024 Jan 16;30(2):403–13. doi: 10.1038/s41591-023-02764-z (PMC10878976; doi:10.1038/s41591-023-02764-z)
Supplement: Supplementary file 1 — Supplementary Table 1. [file 41591_2023_2764_MOESM1_ESM.pdf]

nature medicine

Article

https://doi.org/10.1038/s41591-023-02764-z

Connectivity-guided intermittent theta burst versus repetitive transcranial magnetic stimulation for treatment-resistant depression: a randomized controlled trial

In the format provided by the authors and unedited

Supplementary Table 1. Moderators of primary outcome with TMS

	Number of participants		cgiTBS vs rTMS over 26 weeks	
	rTMS	cgiTBS	Adjusted mean difference (95% CI)	P-value
Moderator analyses				
Baseline GRID-HDRS-17 score	118	115	0.69 (0.45, 0.93)	**0.000**
Baseline GRID-HDRS-17 score*treatment arm			-0.09 (-0.43, 0.24)	0.593
Baseline MGHS score	118	115	0.30 (-0.01, 0.61)	0.058
Baseline MGHS score*treatment arm			0.05 (-0.38, 0.48)	0.830
Age at Baseline	118	115	-0.04 (-0.12, 0.05)	0.391
Age at Baseline*treatment arm			-0.02 (-0.13, 0.09)	0.723
Number of TMS sessions [a]	118	115	-5.64 (-9.48, -1.80)	**0.004**
Number of TMS sessions*treatment arm			3.48 (-2.20, 9.17)	0.230
Baseline CTQ score	111	108	0.004 (-0.06, 0.07)	0.915
Baseline CTQ score*treatment arm			0.01 (-0.08, 0.11)	0.788
Baseline GAD-7 score	118	115	0.36 (0.11, 0.61)	**0.004**
Baseline GAD-7 score*treatment arm			-0.10 (-0.42, 0.23)	0.569
Gender	118	115	0.61 (-1.57, 2.80)	0.581
Gender*treatment arm			-2.19 (-5.32, 0.94)	0.171

Footnote:

[a] This includes number of rTMS or cgiTBS treatment sessions that were delivered as per MRI data or as per changes allowed in the TMS SOP. This moderator was included in the model as a dichotomous variable.

[b] Women vs Men, Men as reference.

Bold indicates statistically significant moderators of outcome.

All analyses were two-sided with no adjustment for multiple comparisons. Moderators regarded as continuous variables with an interaction term between the potential moderator and treatment arm included in the intention to treat analysis using a mixed linear regression model.
