# Supplementary material for: Connectivity-guided intermittent theta burst versus repetitive transcranial magnetic stimulation for treatment-resistant depression: a randomized controlled trial
Source: Nat Med. 2024 Jan 16;30(2):403–13. doi: 10.1038/s41591-023-02764-z (PMC10878976; doi:10.1038/s41591-023-02764-z)
Supplement: Supplementary file 2 — Reporting Summary [file 41591_2023_2764_MOESM2_ESM.pdf]

Corresponding author(s): Richard Morriss
Last updated by author(s): Dec 8, 2023

Reporting Summary

Nature Portfolio wishes to improve the reproducibility of the work that we publish. This form provides structure for consistency and transparency in reporting. For further information on Nature Portfolio policies, see our Editorial Policies and the Editorial Policy Checklist.

Statistics

For all statistical analyses, confirm that the following items are present in the figure legend, table legend, main text, or Methods section.

n/a	Confirmed	
☐	☒	The exact sample size (*n*) for each experimental group/condition, given as a discrete number and unit of measurement
☐	☒	A statement on whether measurements were taken from distinct samples or whether the same sample was measured repeatedly
☐	☒	The statistical test(s) used AND whether they are one- or two-sided<br>*Only common tests should be described solely by name; describe more complex techniques in the Methods section.*
☐	☒	A description of all covariates tested
☐	☒	A description of any assumptions or corrections, such as tests of normality and adjustment for multiple comparisons
☐	☒	A full description of the statistical parameters including central tendency (e.g. means) or other basic estimates (e.g. regression coefficient) AND variation (e.g. standard deviation) or associated estimates of uncertainty (e.g. confidence intervals)
☐	☒	For null hypothesis testing, the test statistic (e.g. *F*, *t*, *r*) with confidence intervals, effect sizes, degrees of freedom and *P* value noted<br>*Give P values as exact values whenever suitable.*
☒	☐	For Bayesian analysis, information on the choice of priors and Markov chain Monte Carlo settings
☒	☐	For hierarchical and complex designs, identification of the appropriate level for tests and full reporting of outcomes
☒	☐	Estimates of effect sizes (e.g. Cohen's *d*, Pearson's *r*), indicating how they were calculated

Our web collection on statistics for biologists contains articles on many of the points above.

Software and code

Policy information about availability of computer code

Data collection	We provide the computer code we used to turn measures of effective connectivity or F3 site from fMRI and structural images into stimulation points for transcranial magnetic stimulation at https://github.com/SPMIC-UoN/brightmind_pipeline
Data analysis	Clinical outcomes were analyzed with STATA version 16 except cognition which was analyzed using SPSS version 25. Neuroimaging employed SPSS version 18 and JASP 018 software.

For manuscripts utilizing custom algorithms or software that are central to the research but not yet described in published literature, software must be made available to editors and reviewers. We strongly encourage code deposition in a community repository (e.g. GitHub). See the Nature Portfolio guidelines for submitting code & software for further information.

Data

Policy information about availability of data

All manuscripts must include a data availability statement. This statement should provide the following information, where applicable:

- Accession codes, unique identifiers, or web links for publicly available datasets
- A description of any restrictions on data availability
- For clinical datasets or third party data, please ensure that the statement adheres to our policy

This is a clinical dataset that will be anonymised and made available as outlined in the manuscript. We used the BrainNet Viewer at www.nitrc.org/projects/bnv/)

with a smoothed brain from the ICBM152 atlas for presentation of brain images. We analysed clinical data with STATA version 16 except for cognition data analyzed with SPSS version 25. Neuroimaging analysis was performed with SPSS version 18 and JASP018 software.

Research involving human participants, their data, or biological material

Policy information about studies with human participants or human data. See also policy information about sex, gender (identity/presentation), and sexual orientation and race, ethnicity and racism.

Reporting on sex and gender	We use the term gender throughout
Reporting on race, ethnicity, or other socially relevant groupings	Age 18 years or over with no upper age limit or limited on gender, ethnicity, marital status or social class.
Population characteristics	Inclusion criteria: Aged ≥18 years old; met criteria for DSM-V major depressive disorder using a structured clinical interview; had moderate to severe depression defined as a score of 16 or more on the GRID version of the 17-Item Hamilton Depression Rating Scale ; had treatment resistant depression defined as scoring 2 or more on the Massachusetts General Hospital Treatment Resistant Depression staging score; and had the capacity to provide informed consent.<br><br>Exclusion criteria: bipolar disorder or depression secondary to other mental disorder; neurological conditions e.g. brain neoplasm, cerebrovascular events, epilepsy, neurodegenerative disorders, prior brain surgery; standard contraindications to magnetic resonance imaging (MRI) (e.g. irremovable metal objects in and around body, pregnancy, red tattoos on the head, neck and back, claustrophobia); major unstable medical illness requiring further investigation or treatment; in two weeks before baseline assessment any change in prescribed medication, treatment with lamotrigine, gabapentin or pregabalin, or intermittent benzodiazepines (or daily prescription above 5 mg diazepam equivalents), hypnotics above 7.5 mg zopiclone equivalent; current substance abuse or dependence (DSM-5 criteria; prior TMS treatment; high risk of suicidality; potential complicating factors for TMS treatment (e.g. hairstyles impeding close coil placement, piercings); involved with any other clinical trial at the time of consent or 6 months prior; unable to read or understand English.
Recruitment	Participants were recruited through specialist mental health services across the five treatment centres In England and neighbouring NHS trusts near the treatment centres, self-referrals, and through patient identification centres recruiting through primary care services. Since this is a sample of treatment resistan depression, then by definition participants must be receiving current treatment form psciliast mental health services or primary care so the results are not applicable to people with moderate to severe depression not receiving treatment. There were more males in the sample but otherwise the sample was demographically representative of treatment resistant depression in England.
Ethics oversight	The clinical trial received research ethics committee approval and health research authority approval from the East Midlands Leicester Central Research Ethics Committee (ref: 18/EM/0232). The research design and execution included local scientists at each site and was shared with all local sites.

Note that full information on the approval of the study protocol must also be provided in the manuscript.

Field-specific reporting

Please select the one below that is the best fit for your research. If you are not sure, read the appropriate sections before making your selection.

☒ Life sciences ☐ Behavioural & social sciences ☐ Ecological, evolutionary & environmental sciences

For a reference copy of the document with all sections, see nature.com/documents/nr-reporting-summary-flat.pdf

Life sciences study design

All studies must disclose on these points even when the disclosure is negative.

Sample size	The National Institute for Health and Care Excellence (NICE) defined 3 points as a clinically important difference in outcome on the HDRS-17 for depression disorders.20 We compared the mean change in depression symptoms from baseline over 26 weeks in the cgiTBS group with that in the rTMS group. Assuming a standard deviation of 8 in the mean difference between groups, as informed by our pilot work19 and a prior randomised controlled trial in chronic persistent depressive disorder,22 a sample size of 266 participants would provide 89.3% power to detect a mean difference of 3 points in the GRID-HDRS-17 over 26 weeks between the groups at the 5% two-sided significance level, assuming a correlation between follow-up measures of 0.7 and 20% data loss/drop-out.
Data exclusions	nclusion criteria: Aged ≥18 years old; met criteria for DSM-V major depressive disorder using a structured clinical interview; had moderate to severe depression defined as a score of 16 or more on the GRID version of the 17-Item Hamilton Depression Rating Scale ; had treatment resistant depression defined as scoring 2 or more on the Massachusetts General Hospital Treatment Resistant Depression staging score; and had the capacity to provide informed consent.<br><br>Exclusion criteria: bipolar disorder or depression secondary to other mental disorder; neurological conditions e.g. brain neoplasm, cerebrovascular events, epilepsy, neurodegenerative disorders, prior brain surgery; standard contraindications to magnetic resonance imaging (MRI) (e.g. irremovable metal objects in and around body, pregnancy, red tattoos on the head, neck and back, claustrophobia); major unstable medical illness requiring further investigation or treatment; in two weeks before baseline assessment any change in prescribed medication, treatment with lamotrigine, gabapentin or pregabalin, or intermittent benzodiazepines (or daily prescription above 5 mg

	diazepam equivalents), hypnotics above 7.5 mg zopiclone equivalent; current substance abuse or dependence (DSM-5 criteria; prior TMS treatment; high risk of suicidality; potential complicating factors for TMS treatment (e.g. hairstyles impeding close coil placement, piercings); involved with any other clinical trial at the time of consent or 6 months prior; unable to read or understand English.
Replication	We did not repeat the experiment to test its reproducibility because it was a large resource intensive multicentre trial with imaging. We have described all procedures so it could be reproduced.
Randomization	We describe the randomisation procedure and how this was communicated
Blinding	Blinding was undertaken and demonstrated

Reporting for specific materials, systems and methods

We require information from authors about some types of materials, experimental systems and methods used in many studies. Here, indicate whether each material, system or method listed is relevant to your study. If you are not sure if a list item applies to your research, read the appropriate section before selecting a response.

Materials & experimental systems

n/a	Involved in the study
☒	☐ Antibodies
☒	☐ Eukaryotic cell lines
☐	☐ Palaeontology and archaeology
☒	☐ Animals and other organisms
☐	☒ Clinical data
☒	☐ Dual use research of concern
☒	☐ Plants

Methods

n/a	Involved in the study
☒	☐ ChIP-seq
☒	☐ Flow cytometry
☐	☒ MRI-based neuroimaging

Palaeontology and Archaeology

Specimen provenance	*Provide provenance information for specimens and describe permits that were obtained for the work (including the name of the issuing authority, the date of issue, and any identifying information). Permits should encompass collection and, where applicable, export.*
Specimen deposition	*Indicate where the specimens have been deposited to permit free access by other researchers.*
Dating methods	*If new dates are provided, describe how they were obtained (e.g. collection, storage, sample pretreatment and measurement), where they were obtained (i.e. lab name), the calibration program and the protocol for quality assurance OR state that no new dates are provided.*

☐ Tick this box to confirm that the raw and calibrated dates are available in the paper or in Supplementary Information.

Ethics oversight	*Identify the organization(s) that approved or provided guidance on the study protocol, OR state that no ethical approval or guidance was required and explain why not.*

Note that full information on the approval of the study protocol must also be provided in the manuscript.

Clinical data

Policy information about clinical studies

All manuscripts should comply with the ICMJE guidelines for publication of clinical research and a completed CONSORT checklist must be included with all submissions.

Clinical trial registration	ISRCTN19674644 complete. CONSORT checklist included
Study protocol	The study protocol has been submitted
Data collection	These are described in the manuscript. All assessments were completed face-to-face at the hospital sites prior to the COVID-19 pandemic, which then changed to video conferencing or telephone methods. Participants also completed a baseline magnetic resonance imaging (MRI) assessment with scans used to derive personalized treatment targets, and for a mechanism of action analysis with MRI at baseline and 16 weeks.
Outcomes	All primary and secondary outcomes are included except health economics data.

Magnetic resonance imaging

Experimental design

Design type	Resting state
Design specifications	Described in the paper. It is a clinical trial
Behavioral performance measures	All the information we required on functional or effective connectivity can be obtained from resting stste fMRI so behavioral performance measures were not necessary.

Acquisition

Imaging type(s)	functional MRI
Field strength	3Tesla
Sequence & imaging parameters	Multimodal MRI at 3T consisting of a structural T1-weighted scan and an eyes-open blood oxygenation level dependent (BOLD) echo-planar imaging (EPI) resting state fMRI scan with additional positive and negative phase-encoded images to enable distortion correction.High-resolution T1-weighted images will be acquired using sagittal fast-spoiled gradient echo BRAVO (or equivalent) sequences with 1 mm3 isotropic voxels covering the whole head from the vertex to the neck. rsfMRI images will be acquired with the eyes open using a fixation cross. All sites used a gradient echo EPI sequence aligned with the anterior commissure-posterior commissure line, with acquisition covering from the vertex downward (repetition time [TR]/echo time [TE]=2000/32 ms; flip angle=77°; 35 slices; voxel size=3 mm3; slice gap=0.5 mm; field of view=192×192 mm; interleaved bottom/up; 240 volumes; phase encoding direction=posterior>anterior). All rsfMRI images have associated forward- and reverse-phase-encoded B0 images acquired to facilitate distortion correction.
Area of acquisition	Whole brain

Diffusion MRI ☐ Used ☒ Not used

Preprocessing

Preprocessing software	Subject digital imaging and communications in medicine (DICOM) session files were uploaded onto an XNAT (Washington University School of Medicine) database infrastructure for all data other than MRS data using anonymized subject numbers. Once the session was archived within XNAT, it was put into a quarantined state awaiting quality control (QC), and DICOM files were automatically converted into Brain Imaging Data Structure NIFTI or JSON pairs for each scan using the dcm2bids-session v1.5 XNAT container, with T1-weighted images also undergoing defacing within this step.
Normalization	Normalization.Structural T1-weighted images were first coarsely brain extracted using the FSL brain extraction tool (BET). The original and brain extracted images are then nonlinearly registered to the MNI152 1-mm template using FSL FMRIB's nonlinear image registration tool (FNIRT). The original FSL BET brain extraction was then refined by applying the produced nonlinear transformation to warp the MNI152 brain mask onto the subject's T1 image. The resulting brain extracted image was finally bias-corrected and segmented into gray matter, cerebrospinal fluid (CSF), and white matter (WM) using FSL FMRIB Automated Segmentation Tool (FAST). The resulting WM and CSF probability maps were binarized at a tissue-probability threshold of 98% and then eroded using a spherical kernel with a radius of 2 voxels.
Normalization template	Normalization template. The MNI152 1-mm template using FSL FMRIB's nonlinear image registration tool (FNIRT).
Noise and artifact removal	Noise and artifact removal.The structural T1-weighted image was bias-corrected after nonlinear transformation. BOLD rsfMRI images underwent EPI distortion correction by inputting the positive and negative phase-encoded acquisitions into TOPUP. Then, they underwent between-volume motion correction (MCFLIRT 6DoF) and SPM12 interleaved slice-timing correction (bottom-up). The corrected BOLD image was subsequently smoothed with a 5 mm full-width half-maximum kernel using Smallest Univalue Segment Assimilating Nucleus (SUSAN) and denoised with ICA-AROMA. BOLD images were high-pass filtered at a frequency of 0.01 Hz after denoising. A transformation between the resulting BOLD image and the T1-weighted image was later computed using FSL epi_reg, and then combined with the TOPUP spatial distortion correction transformation. The resulting combined transformation was then inverted to create a nonlinear transformation from the T1-weighted to (original uncorrected) BOLD space. The previously computed binary WM and CSF masks were later warped into BOLD space using the T1-weighted to BOLD transformation to extract the WM and CSF time series from the BOLD data. To control for additional physiological or scanner-related noise, the WM and CSF time series were then regressed out of the rsfMRI time series.
Volume censoring	The field of view was reduced by removing the lower head and neck using FSL robustfov.

Statistical modeling & inference

Model type and settings	Model type and settings.Functional connectivity (FC). Time series was extracted from two regions of interest (ROIs) using the fslmeants tool (part of the FSL software library). The first principal component of the time series of all voxels within an ROI was taken as a representation of the overall time series for that ROI. The first five time points were discarded to allow magnetisation stabilisation and time series were band-pass filtered between 0.01 and 0.1 Hz. Zero-lag ROI-to-ROI

correlations were calculated using Pearson correlations between the times series, partialling out twenty-four head motion parameters (three translations and three rotations for current time point and one time point prior, and squares of these parameters). Correlations of interest were converted to z-scores using Fisher's r-to-z conversion. These z-scores served as the measures of FC.
Effective connectivity (EC) Granger causality was computed using the REST toolbox, which runs under MATLAB. This provided a measure of directed (effective) connectivity, from one ROI (x, for example, right AI) to another ROI (y, for example, left DLPFC). The toolbox provides measures of the influence of x on y as well as y on x. The first five time points were discarded, and the twenty-four head motion parameters were entered as covariates. No band-pass filter was applied, as per the trial target-identification procedure. Effective connectivity values we converted to z-scores using Fisher's r-to-z conversion, then the mean z-score output across all voxels in y served as the measure of EC.

Effect(s) tested

A map of left DLPFC effective connectivity from rAI was first calculated. The maximum of this map corresponds to the cgiTBS target. The ROI for left DLPFC was a 6-mm sphere centred on the cgiTBS target co-ordinates themselves (regardless of treatment group). the ROI for right AI was a 6-mm sphere centred on MNI co-ordinates x=30, y=24, z=-14. We calculated effective connectivity as a function of distance from this maximum to examine whether optimal targets according to the trial target identification procedure were clearly unique or whether there are multiple, separate, potential targets within DLPFC. For each participant, we computed functional connectivity between each image voxel and 6-mm spherical seed regions centred on the intended stimulation target co-ordinates. We computed these seed-region functional connectivity images for baseline and follow-up, and also computed the difference between the follow-up and baseline images.
To examine DLPFC-DMPFC connectivity, two centroids for the left DLPFC ROI that are independent from the cgiTBS and rTMS co-ordinates were examined in separate analyses: an anterior DLPFC centroid, x = -44, y = 40, z = 29, and a more posterior DLPFC centroid, x = -44, y = 22, z = 36. The centroid for the left DMPFC ROI was x = -7, y = 49, z = 18.

Specify type of analysis: ☐ Whole brain ☒ ROI-based ☐ Both

Anatomical location(s)

Describe how anatomical locations were determined (e.g. specify whether automated labeling algorithms or probabilistic atlases were used).

Statistic type for inference

(See Eklund et al. 2016)

Statistic type for inference. Mixed effects models were implemented in SPSS (version 18) and JASP (0.18) software and estimated with restricted maximum likelihood. Participant served as the random effect with a scaled identity variance-covariance matrix, and the dependent variable was clinical improvement from baseline in GRID-HDRS-17 (primary outcome measure), PHQ-9 or BDI-II (planned exploratory outcome measures). As well as the baseline connectivity, or change in connectivity, relevant to a given hypothesis, we included as independent variables the post-treatment time point (8, 16, 26 weeks) and treatment group (rTMS, cgiTBS), and the interaction of connectivity with either or both variables. Age, gender, MGH treatment resistance group, GAD-7, CTQ and study group site were explored as potential confounding variables: where these were non-significant, they were removed from the model.

Correction

Bonferroni for functional connectivity between left dorsolateral prefrontal cortex to left dorsomedial prefrontal cortex only.

Models & analysis

n/a	Involved in the study
☐	☒ Functional and/or effective connectivity
☒	☐ Graph analysis
☒	☐ Multivariate modeling or predictive analysis

Functional and/or effective connectivity

Functional and effective connectivity were used as outlined under model type and settings.
